# Hyperpolarized ^13^C Spectroscopy with Simple Slice-and-Frequency-Selective Excitation

**DOI:** 10.3390/biomedicines9020121

**Published:** 2021-01-27

**Authors:** Geoffrey J. Topping, Irina Heid, Marija Trajkovic-Arsic, Lukas Kritzner, Martin Grashei, Christian Hundshammer, Maximilian Aigner, Jason G. Skinner, Rickmer Braren, Franz Schilling

**Affiliations:** 1 Department of Nuclear Medicine, School of Medicine, Klinikum Rechts der Isar, Technical University of Munich, 81675 Munich, Germany; geoff.topping@tum.de (G.J.T.); martin.grashei@tum.de (M.G.); christian.hundshammer@wacker.com (C.H.); maximilian.aigner@tum.de (M.A.); jason.skinner@tum.de (J.G.S.); 2 Institute of Diagnostic and Interventional Radiology, School of Medicine, Klinikum Rechts der Isar, Technical University of Munich, 81675 Munich, Germany; irina.heid@tum.de (I.H.); lukas.kritzner@tum.de (L.K.); rbraren@tum.de (R.B.); 3 Division of Solid Tumor Translational Oncology, German Cancer Consortium (DKTK, Partner Site Essen), 45147 Essen, Germany; m.trajkovic-arsic@dkfz-heidelberg.de; 4 German Cancer Research Center, DKFZ, 69120 Heidelberg, Germany; 5 Institute of Developmental Cancer Therapeutics, West German Cancer Center, University Hospital Essen, 45147 Essen, Germany; 6 German Cancer Consortium (DKTK, Partner Site Munich), 81675 Munich, Germany

**Keywords:** magnetic resonance slice spectroscopy, narrow bandwidth excitation, point resolved spectroscopy, hyperpolarized ^13^C lactate, rat subcutaneous tumor

## Abstract

Hyperpolarized ^13^C nuclear magnetic resonance spectroscopy can characterize in vivo tissue metabolism, including preclinical models of cancer and inflammatory disease. Broad bandwidth radiofrequency excitation is often paired with free induction decay readout for spectral separation, but quantification of low-signal downstream metabolites using this method can be impeded by spectral peak overlap or when frequency separation of the detected peaks exceeds the excitation bandwidth. In this work, alternating frequency narrow bandwidth (250 Hz) slice-selective excitation was used for ^13^C spectroscopy at 7 T in a subcutaneous xenograft rat model of human pancreatic cancer (PSN1) to improve quantification while measuring the dynamics of injected hyperpolarized [1-^13^C]lactate and its metabolite [1-^13^C]pyruvate. This method does not require sophisticated pulse sequences or specialized radiofrequency and gradient pulses, but rather uses nominally spatially offset slices to produce alternating frequency excitation with simpler slice-selective radiofrequency pulses. Additionally, point-resolved spectroscopy was used to calibrate the ^13^C frequency from the thermal proton signal in the target region. This excitation scheme isolates the small [1-^13^C]pyruvate peak from the similar-magnitude tail of the much larger injected [1-^13^C]lactate peak, facilitates quantification of the [1-^13^C]pyruvate signal, simplifies data processing, and could be employed for other substrates and preclinical models.

## 1. Introduction

Hyperpolarized ^13^C magnetic resonance spectroscopy and spectroscopic imaging are used to characterize metabolism in vivo using a variety of acquisition schemes [1,2,3], both in preclinical studies [4,5,6] and clinical settings [7,8]. An important application area of spectroscopic imaging with hyperpolarized [1-^13^C]pyruvate is detection of metabolic changes of inflammatory processes caused by infiltrating immune cells such as macrophages. For example, increased levels of [1-^13^C]lactate were noted in murine models of multiple sclerosis caused by an invasion of pro-inflammatory mononuclear phagocytes [9] as well as in complete Freund’s adjuvant (CFA)-induced inflammatory arthritis [10] and lipopolysaccharide (LPS)-induced neuroinflammation in the brain [11]. Rather than being considered a waste product of metabolism, lactate is being recognized as actively modulating the inflammatory immune response and is therefore an attractive target for molecular imaging [12].

A relatively simple method for time-resolved “dynamic” ^13^C spectroscopy is broad bandwidth slice-selective excitation and free-induction decay (FID) spectral readout [13]. For [1-^13^C]pyruvate (171 ppm) to [1-^13^C]lactate (183 ppm) conversion [14,15,16], spectra are typically acquired every 1 to 3 s and may be analyzed to extract biomarkers involving kinetic rate constants of conversions between these metabolites [17,18].

More challenging are compounds and labels with wider frequency separation, like [^13^C]HCO_3_^−^ (162 ppm) and [^13^C]CO_2_ (124 ppm) [17,19], or [2-^13^C]pyruvate (208 ppm), [2-^13^C]lactate (71 ppm), and [2-^13^C]alanine (53 ppm) [20,21,22]. Widely spread chemical shifts may cause slice displacement artifacts, which could cause lower signal intensities or inconsistent slice positions between metabolites, or which may require prohibitively wide transmit bandwidth to simultaneously excite.

Substrates with lower levels of the downstream metabolites are also challenging, like conversion of [1-^13^C]lactate into [1-^13^C]pyruvate and [^13^C]HCO_3_^−^ [6,23], particularly when chemical shifts of metabolites [20] are close. For injected [1-^13^C]lactate and downstream [1-^13^C]pyruvate with broad bandwidth excitation, the tails of the injected lactate peak are comparable in magnitude to the pyruvate peak at its central frequency, potentially interfering with pyruvate quantification [6].

This work addresses the quantification of [1-^13^C]pyruvate after conversion from [1-^13^C]lactate in a subcutaneous xenograft rat model of human pancreatic cancer (PSN1). An alternating frequency [21,24,25] narrow bandwidth slice-selective [26] excitation strategy is combined with point-resolved spectroscopy of the thermal ^1^H signal in the target region for ^13^C frequency calibration [27], in order to ensure accurate slice positioning for ^13^C according to the prescription on ^1^H anatomical images.

## 2. Experimental Section

### 2.1. Subjects and Tumors

Rats (2 male and 1 female, Crl:NIH-Foxn1^rnu^, Charles River) were implanted subcutaneously in the flank with 10^7^ PSN1 cells dissolved in 100 µL of PBS. Cells were cultured in Dulbecco’s modified Eagle Medium (DMEM) containing L-glutamine (Biowest, Nuaillé, France), 10% fetal calf serum (Merck, Darmstadt, Germany), 1% sodium pyruvate (Merck, Darmstadt, Germany), 1% non-essential amino acids (GE Healthcare, Chicago, IL, USA) and 1% penicillin and streptomycin (PAN Biotech, Aidenbach, Germany) at 37 °C with 5% CO_2_. Tumors grew to 10 mm in length before hyperpolarized measurements.

Rats were anesthetized with isoflurane 2.5% (*v*/*v*) in an oxygen flow rate of 2 L/min. Breathing rates (50–70 bpm) and temperature (37–39 °C) were monitored. Hyperpolarized substrates (1.5 mL, 80 mM for [1-^13^C]pyruvate and 100 mM for [1-^13^C]lactate) were injected via tail vein catheter approximately 20 s after dissolution.

Animal protection and welfare review board approval was received prior to study initiation (Regierung von Oberbayern, Munich, Germany, Approval Number ROB-55.2- 2532.Vet_02-18-92 from 10.01.2019). All experiments were carried out in adherence to pertinent laws and regulations.

### 2.2. Imaging System

**Figure 1:**
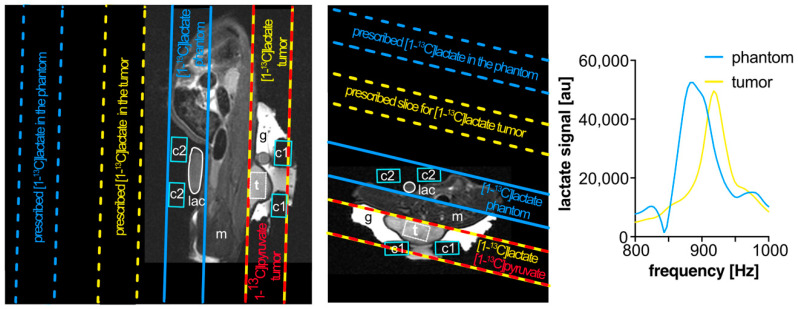
Representative anatomical T2-weighted (rapid acquisition with relaxation enhancement (RARE)) images in sagittal (**A**) and axial (**B**) orientations, with oblique spectroscopy slice geometry shown for a subcutaneous tumor (**t**) in a rat and for a thermal lactate (**lac**) phantom for transmit power calibration. To measure within the target tumor, with the scanner reference frequency set to that of [1-^13^C]pyruvate, the slice geometry is prescribed twice: once on the target tumor for [1-^13^C]pyruvate (yellow-and-red outlines) and again offset by the chemical shift displacement of [1-^13^C]lactate (dashed yellow outlines). This produces excitations that, at the location of the tumor, alternate between [1-^13^C]pyruvate and [1-^13^C]lactate frequencies. An additional slice may also be prescribed (dashed blue outlines) to target lactate in the phantom (solid blue outlines). Carbopol^®^ 980 gel (**g**, bright contrast) was placed around the tumor to improve its B0 field uniformity. The locations of the ^13^C surface receive coils (**c1** and **c2**, light blue outlines), muscle (**m**), and a point-resolved spectroscopy (PRESS) voxel (white box surrounding **t**) for shimming on proton signal within the tumor are also marked. Magnitude spectra (**C**) from [1-^13^C]lactate in the phantom (blue) and from hyperpolarized [1-^13^C]lactate in the tumor (yellow) after injection, exhibiting a frequency shift between the two sources of lactate signal. In cases where this shift is large, the placement of a slice prescribed to measure within the phantom may benefit from additional spatial offset to compensate for that frequency shift in order to excite the ^13^C in the phantom.

Images and spectra were acquired using a small animal 7 T preclinical scanner (Agilent Discovery MR901 magnet and gradient system, Bruker AVANCE III HD electronics) with a dual-tuned ^1^H/^13^C volume resonator (inner diameter 72 mm, RAPID Biomedical) for proton anatomical imaging, shimming, and frequency calibration, and for ^13^C excitation. Surface receiver coils (20 mm diameter, RAPID Biomedical) were placed on top of the tumors and near a thermally-polarized [1-^13^C]lactate phantom [28] (for pre-scan adjustments) for ^13^C signal reception. The signals from the two receiver coil channels were processed separately for the area adjacent to the coils. Carbopol^®^ 980 gel (Caesar & Loretz GmbH, Germany) surrounded the tumor for improved B0 shim uniformity (Figure 1).

### 2.3. Proton Imaging and ^13^C Pre-Scan Adjustments

**Figure 2:**
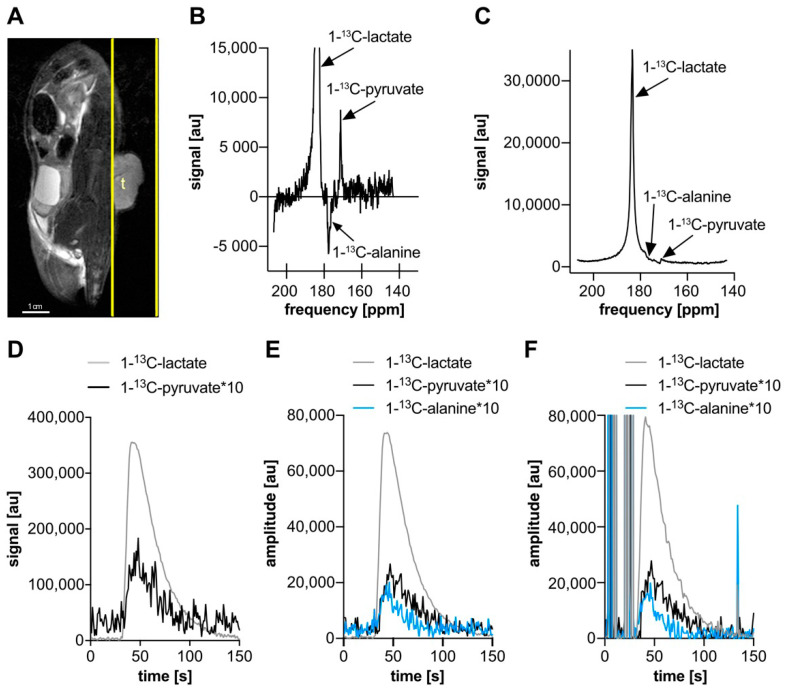
Broad bandwidth (5 kHz) excitation slice spectroscopy in a rat subcutaneous tumor after hyperpolarized [1-^13^C]lactate injection. (**A**) Representative sagittal T2-weighted (RARE) anatomical image of rat with tumor (**t**) and slice geometry (yellow outline). (**B**,**C**) Spectra summed over 13 frames (26 s) covering the [1-^13^C]pyruvate signal maximum. (**B**) Real part of manually phased complex spectrum with zoomed signal range to show smaller peaks and background. The lactate peak is narrower than in (**C**), but determining phase factors to remove background signal at the alanine and pyruvate peak center frequencies is difficult, affecting their quantification. (**C**) Magnitude spectrum in which the tail of the lactate spectral peak (centered near 183.5 ppm) is comparable in magnitude to the pyruvate (near 171 ppm) and [1-^13^C]alanine (near 177 ppm) at their central frequencies, and the peaks interfere with each other, distorting the shape of the lactate peak tail. (**D**) Time course of the lactate and pyruvate spectral peak magnitudes, before and after lactate injection (near 30 s). (**E**,**F**) Time course of AMARES lactate, alanine, and pyruvate fit magnitudes with highly (**E**) and lightly (**F**) constrained models. Early time-point fits before lactate injection have implausible and highly variable amplitudes in the lightly constrained model. Between these models, the lactate time course appears different near its peak, and the alanine and pyruvate curves appear to have a higher mean value at later times (> 90 s) in the highly constrained fit.

Anatomical proton images (Figure 1 and Figure 2A) were acquired using a multi-slice T2-weighted rapid acquisition with relaxation enhancement (RARE) sequence, to guide placement of spectroscopy voxels and slices on the tumor. Typical imaging parameters were echo time TE = 30 ms, repetition time TR = 3 s, field of view 128 × 72 mm^2^, in-plane resolution 0.5 × 0.5 mm^2^, 29 slices of 2 mm thickness, and 3 image averages.

B0 maps, acquired using the scanner’s FieldMap (3D double gradient recalled echo) sequence, were used to set 2nd order shim currents for the tumor region. A point-resolved spectroscopy (PRESS) voxel on the tumor was used for additional localized iterative shim adjustment [29] and to set the proton center frequency, which was scaled to the frequency for [1-^13^C]pyruvate [27].

Transmission gain (reference power) for ^13^C was calibrated with a thermally-polarized [1-^13^C]lactate phantom [28] placed on the opposite side of the body from the tumor (Figure 1). FIDs from a spatially non-selective sequence with 1 ms radiofrequency (RF) block pulses and incrementing power were analyzed in MatLab with custom-written scripts. The central frequency for this adjustment was separately calibrated, using thermal signal from the phantom itself.

### 2.4. Polarization

Samples containing ^13^C-labeled compounds (Merck, Darmstadt, Germany) were polarized, with either 1) 14 M [1-^13^C]pyruvate and 1 mM Dotarem in ddH_2_O or 2) 3.1 M [1-^13^C]sodium-lactate in 30% (*v*/*v*) DMSO and 70% ddH_2_O. Both preparations also contained 15 mM OX063 trityl radical. Pyruvate and lactate were hyperpolarized for 45 min and 180 min, respectively, with a HyperSense^®^ DNP Polarizer (Oxford Instruments, Abingdon, UK) at 1.2 K and 3.35 T, with a microwave frequency of 94.19 GHz and 100 mW power. Pyruvate was dissolved in 3.4 ± 0.3 mL buffered solution that was heated to 180 °C and contained 80 mM TRIS, 0.1 g/l EDTA and 80 mM sodium hydroxide (NaOH), resulting in a 80 mM [1-^13^C]pyruvate solution with mean pH of 7.1 ± 0.2. Lactate [6,30,31] was similarly dissolved in 3 mL of 80 mM TRIS/0.1 g/l EDTA/D_2_O/1 M NaOD solution, resulting in a 100 mM [1-^13^C]lactate solution, with mean pH of 7.3 ± 0.2.

### 2.5. Slice-Selective ^13^C Spectroscopy

Broad bandwidth slice-selective spectroscopy used Shinnar–Le Roux (SLR) excitation RF pulses (5 kHz full width at half maximum (FWHM) bandwidth, 0.68 ms duration, sharpness 3, flip angle 5°), centered between the [1-^13^C]lactate and [1-^13^C]pyruvate resonances (175 ppm). The sequence had repetition time of 2 s, readout bandwidth of 2 kHz, 512 FID point readout, and single 15 mm-thick slices placed over tumors (Figure 2A).

Narrow bandwidth slice-selective spectroscopy also used SLR excitation pulses (250 Hz FWHM bandwidth, sharpness 1, duration 8.4 ms, flip angle 30°), alternating between metabolite frequencies [21]. At 920 Hz off-resonance—the frequency difference between [1-^13^C]lactate and [1-^13^C]pyruvate at 7 T—this pulse shape produces under 0.05% of the on-resonance flip angle (using a small flip angle approximation).

To implement alternating frequency excitation of [1-^13^C]lactate and [1-^13^C]pyruvate at the same slice position, without support in the pulse sequence for variable transmission reference frequency or specialized spectral–spatial excitation pulses, two 15 mm-thick slices were prescribed, exploiting the chemical shift displacement artifact [20]. This displacement depends on the slice-selection gradient, or equivalently, the slice thickness and RF excitation bandwidth. For consistent definitions thereof [26],

(1) $$\mathrm{Slice}\;\mathrm{Offset}\;=\frac{\mathrm{Slice}\;\mathrm{Thickness}\;\times \;\mathrm{Frequency}\;\mathrm{Offset}}{\mathrm{RF}\;\mathrm{Pulse}\;\mathrm{Bandwidth}}=\frac{\mathrm{Frequency}\;\mathrm{Offset}}{\mathrm{Gradient}\;\times \;\gamma }$$

where *γ* is the gyromagnetic ratio of the nucleus (here ^13^C) being excited.

With the reference frequency set for [1-^13^C]pyruvate, slices were placed 1) on the tumor and 2) outside of the body on the far side of the tumor at a relative slice position offset of 55.2 mm (Figure 1). This offset corresponds to the chemical shift displacement of [1-^13^C]lactate from [1-^13^C]pyruvate at 7 T for 15 mm slice thickness and 250 Hz transmit bandwidth (16.67 Hz/mm gradient and 920 Hz frequency offset). This sequence had two excitations per total repetition time (2 s), readout bandwidth (2 kHz), 512 FID points per excitation, and was used for both injections of hyperpolarized [1-^13^C]lactate and [1-^13^C]pyruvate.

### 2.6. Spectral Analysis

Broad bandwidth spectral component time courses were estimated from the signal magnitudes at the frequencies of [1-^13^C]lactate and (approximately) [1-^13^C]pyruvate (Figure 2C,D), which were determined from the spectrum after averaging over a period covering the lactate signal peak. This averaged complex spectrum was also manually phased (0th and 1st order factors) and its real part was plotted (Figure 2B).

The AMARES algorithm [32], available in the jMRUI program [33,34], was also applied to isolate the [1-^13^C]pyruvate, [1-^13^C]alanine, and [1-^13^C]lactate components. Several AMARES models were applied to the data set shown in Figure 2, which all allowed the Lorentzian peak amplitudes and phases to freely and independently vary between frames, with other parameters either fixed or allowed to vary freely between frames:

1. Flexible: free peak frequencies, free peak widths, free zeroth order phase, and free begin time (1st order phase);
2. Lightly constrained: fixed peak frequencies (8.41, 2.37, and 3.97 ppm relative to the ^13^C reference frequency), free lactate peak width, alanine and pyruvate peak widths fixed to that of lactate in each frame, free zeroth order phase, and free begin time;
3. Moderately constrained: fixed frequencies (as in model 2), fixed peak line widths (54 Hz, based on the lightly constrained lactate peak width results in frames with the largest lactate signal), free zeroth order phase, and free begin time;
4. Highly constrained: fixed frequencies (as in model 2), fixed peak widths (as in model 3), fixed zeroth order phase and begin time (50.1° and 9.8 ms, based on separate fit to sum of 69 frames covering the peak with the same model except those two parameters free).

Ratios of the areas under the curves (AUCr) of pyruvate and lactate were calculated, starting from the frame before lactate signal appears and summing until the end of acquired data, in order to compare the results of the different AMARES fits and the magnitude spectral plot of the same data and similarly acquired narrow bandwidth data. For the broad bandwidth magnitude spectra, the magnitude for each metabolite was taken at a frequency where the peaks appeared centered in the manually phased real spectrum, which were separated by a distance consistent with the spacing of the fixed peak frequencies that were used in the AMARES fitting with constrained models.

## 3. Results

### 3.1. Frequency Calibration

A thermal [1-^13^C]lactate phantom near the subject (Figure 1) was inadequate for frequency calibration [28] for narrow bandwidth excitation because the resulting frequency was often not accurate for the tumor location. A PRESS voxel measuring the local ^1^H frequency was easily placed and used for adjusting the ^13^C frequency at the same location, based on the chemical shift offset for [1-^13^C]pyruvate.

### 3.2. Broad Bandwidth Spectra

Broad bandwidth excitation slice spectroscopy of a subcutaneous tumor after hyperpolarized [1-^13^C]lactate injection showed strong [1-^13^C]lactate and weak [1-^13^C]pyruvate signal (Figure 2). In magnitude spectra near the peak pyruvate signal amplitude (Figure 2C,D), the lactate peak has a broad tail that extends to the pyruvate central frequency, impairing pyruvate quantification due to interference between peaks. In the range between the alanine and pyruvate central frequencies, the spectrum is lower than the opposite side of the lactate tail at the same frequency offsets from the lactate peak (where other interfering peaks are not observed). In phased real spectra (Figure 2B), the lactate tail is narrower and the pyruvate peak can be resolved, but reliably phasing such spectra, automatically or manually, is difficult given the sensitivity of small peak heights to shifts in the spectral baseline signal. At the pyruvate central frequency, near the peak pyruvate signal (Figure 2C), the amplitude of the pyruvate peak is less than the lactate tail magnitude at the same frequency offset on the other side of the lactate peak.

### 3.3. Spectral Fitting

The AMARES flexible model fit to the broad bandwidth excitation spectra failed almost completely for the pyruvate and alanine frequencies, with most time frames having completely implausible amplitudes and large inconsistencies between adjacent frames. The AMARES fitting with the light, moderate, and highly constrained models generally produced similarly shaped time courses, but different AUCr for both pyruvate/lactate and alanine/lactate (Table 1). The less-constrained models had lower AUCr (pyruvate/lactate or alanine/lactate) than more constrained models. The lightly constrained model (Figure 2F) also had numerous frames in which the fit failed and produced strongly inconsistent amplitudes compared with adjacent frames, particularly in frames before the lactate injection occurred, whereas the moderate and highly (Figure 2E) constrained models did not have these obvious fit failures. The peak width from the lightly constrained model, which allowed peak width to vary between frames but was kept the same for all three fit metabolites, varied with frame, starting near 60 Hz at frame 33 and dropping to about 53 Hz by frame 56, and was thereafter increasingly variable between frames but tended to be more stable on average with increasing frames. Overall, with increasingly constrained fits, the results were more stable and consistent between frames.

### 3.4. Narrow Bandwidth Spectra

**Figure 3:**
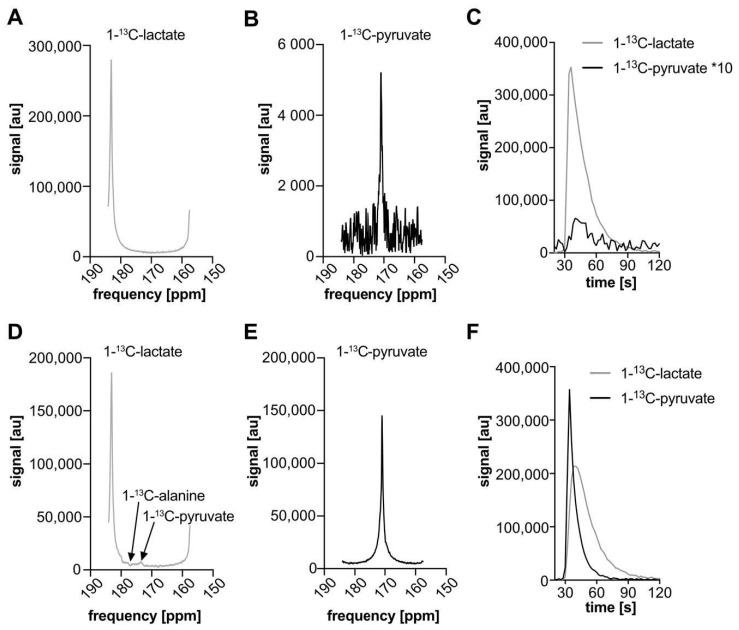
Magnitude spectra (**A**,**B**,**D**,**E**) and time courses (**C**,**F**) of hyperpolarized [1-^13^C]pyruvate (**B**,**E**) and [1-^13^C]lactate (**A**,**D**) acquired with narrow bandwidth (250 Hz) alternating-frequency excitation slice-selective spectroscopy in rat subcutaneous tumors at 7 T (representative results of one rat out of three measurements). Spectra were summed over 15 frames (30 s) surrounding the peaks. After [1-^13^C]lactate injection (**A**–**C**), a strong lactate signal (near 183.5 ppm) and weak [1-^13^C]pyruvate signal (near 171 ppm) are seen. The pyruvate-selective excitation has eliminated the background signal tail from the lactate peak (Figure 2C). After [1-^13^C]pyruvate injection (**D**–**F**), both pyruvate and lactate show strong signal with narrow bandwidth excitation. Small ripples in (**D**) correspond to [1-^13^C]alanine and [1-^13^C]pyruvate excited in the body of the rat by the lactate-targeted excitation, at shifted frequencies due to shim variations outside the tumor.

After hyperpolarized [1-^13^C]lactate injection, narrow bandwidth excited lactate was seen after its own narrow bandwidth excitation (Figure 3A) and [1-^13^C]pyruvate was detected without lactate tail background (Figure 3B,C). The AUCr for pyruvate/lactate was lower compared to the values obtained by AMARES fitting to the broad bandwidth excited spectra (Table 1). A similar experiment with hyperpolarized [1-^13^C]pyruvate also detected both peaks without substantial background signals (Figure 3D-F), at the targeted frequencies. The [1-^13^C]lactate excitation after [1-^13^C]pyruvate also shows small deviations in the spectra, similar to Figure 2C, from co-excited [1-^13^C]alanine and [1-^13^C]pyruvate (Figure 3E), but these do not interfere with lactate quantification.

## 4. Discussion

Chemical shift displacement artifacts and low signal-to-noise ratio (SNR) metabolite signals in the presence of higher background signals are challenges for the quantification of metabolites using slice-selective excitation. This work demonstrates a strategy for acquiring narrow bandwidth excited slice spectroscopy of hyperpolarized [1-^13^C]lactate and [1-^13^C]pyruvate in rat subcutaneous tumors, with ^13^C frequency calibration based on proton signal from PRESS voxels on the target regions. The combination of these techniques improves slice selective acquisition and eliminates spectral background signal from a large spectral peak near a much smaller peak of interest.

This work uses the metabolite chemical shift displacement slice offset to implement alternating frequency slice-selective excitation [26]. This method may be used when the metabolites of interest have fixed chemical shifts that are known prior to the measurement. Slices can be positioned in this manner with neither alternating-frequency excitation and explicit specification of frequency offsets, nor associated alternating gradient polarity [26], being nominally supported by the pulse sequence. This method also does not require careful design of intermediate bandwidth RF pulses to place pass and stop bands relative to metabolite frequencies [21] or advanced spectral–spatial RF-and-gradient pulses [35,36,37], which are less likely to be available on all MRI systems without substantial development work.

Frequency calibration for ^13^C from ^1^H tumor-local signal was measured in this work with a PRESS voxel placed on the tumor. This avoids the need for direct ^13^C frequency calibration, which would either waste limited hyperpolarized signal, or which would require using signal from a separate thermal ^13^C phantom. Such a phantom-derived frequency calibration is sufficient for a broad bandwidth excitation spectroscopy measurement, as shifts of even hundreds of Hz should not substantially affect the flip angle distribution from a reasonably-flat-response 5000 kHz bandwidth excitation. However, for narrow bandwidth (250 Hz) excitation, more precise frequency calibration at the tumor location is necessary to ensure accurate slice positioning for ^13^C, consistent with the prescription on ^1^H anatomical images. Frequency calibration for ^13^C based on the average ^1^H frequency across the subject [27] likely has similar limitations for accuracy at the location of a subcutaneous tumor, although it would be more suitable for imaging internal organs, where B0 varies less than at the surface of the body, and thus shimming is less difficult and where the frequency is likely to be more consistent with the overall average of the imaged volume.

A potential problem with narrow bandwidth excitation is distortion of the excited slice geometry. Due to a combination of subject-induced B0 variation and second-order shim gradients that are applied to minimize the peak width within a superficial tumor, the B0 field may be non-linearly distorted away from the target location. When using very narrow bandwidth excitation and thick slices (which are necessary to ensure the chemical shift offset places undesired metabolite excitations far from the target region and outside the body), these field variations may be large relative to linear slice-selection gradients. However, in this application, this issue is mitigated by the use of a surface receiver coil placed on top of the target region and by imaging low-γ ^13^C nuclei. Even if the slice geometry is distorted in such a way as to bend back into the subject’s body, rather than adhering to the prescribed slice geometry that intersects only the tumor, the surface coil will be sensitive primarily and most strongly near its location, and will be minimally sensitive to any magnetization excited elsewhere in the body.

More generally, the reproducibility of the proposed narrow bandwidth excitation scheme is likely dominated by operator-dependent and biological factors in a practical measurement. The sequence itself should be perfectly reproducible, outside of hardware or software failures in the scanner. Operator-dependent factors include positioning of animals, placement of gel around tumors, placement of receiver coils, placement of shim and frequency calibration volumes, excitation slice prescription, and the timing of hyperpolarized injections. Biological factors include details of anesthesia and temperature control, timing of previous injections, and tumor metabolism, perfusion, and growth. Some of these aspects could be tested in isolation, such as by repeatedly prescribing shim volumes and frequency calibration voxels, but their impact on the reproducibility results of the subsequent hyperpolarized measurement is difficult to experimentally and meaningfully assess. Measuring the same or different tumors or different days will lead to substantial variations in results, even if all operator-dependent factors were very consistent. These factors render statistical testing of reproducibility of biologically dependent outcomes against similar methods impractical.

Broad bandwidth excitation FID readout spectroscopy is well suited for measuring the metabolism of hyperpolarized substrates and their downstream metabolites because it quantitatively captures several hyperpolarized molecules at once, to follow metabolic processes. Usefully, the frequencies of metabolites do not need to be precisely known prior to measurement. However, it requires relatively narrow chemical shift separation between the peaks, because achieving a single RF pulse with a nearly equal flip angle for multiple metabolites is limited by the peak RF power transmission.

In recent years, narrow bandwidth excitation been employed for single-frame imaging of hyperpolarized [1-^13^C]pyruvate and its metabolites in mouse kidneys [26] and multi-frame imaging of [2-^13^C]pyruvate and its metabolites in rat hearts [21]. In this work, the injected hyperpolarized [1-^13^C]lactate produces much less downstream [1-^13^C]pyruvate than the reverse process, and the subcutaneous tumor target region has generally poorer delivery of a hyperpolarized agent than the well-perfused internal organs do. These factors limit the practical measurement to non-imaging spectroscopy, as demonstrated in this work, and make the narrow bandwidth excitation approach particularly beneficial to improve quantification of the smaller metabolite signal.

Narrow bandwidth excitation is also particularly useful for systems with large differences in magnitude of peaks that are closely spaced in frequency, particularly for dynamic measurements. With well-separated or similar-magnitude peaks, the signals are more easily quantified after broad bandwidth excitation. With dynamic measurements, advanced spectral fitting algorithms can be unreliable, making spectral signal isolation during acquisition more advantageous. Metabolic processes, like conversion of [1-^13^C]lactate to [1-^13^C]pyruvate and [1-^13^C]bicarbonate [6] or [1-^13^C]glucose to [1-^13^C]glycogen [38], are thus potential applications for narrow bandwidth methods.

Reliable signal quantification with broad bandwidth excitation hyperpolarized ^13^C spectroscopy may be difficult when a small peak overlaps with the tails of a nearby larger peak. Complex spectra may be used to determine peak amplitudes, and can substantially reduce the impact of interference between close-by metabolite signals because peaks are narrower than in magnitude spectra. However, the tails of a much larger peak can still be non-negligible at the location of a smaller peak, and phasing spectra can be difficult with low signal-to-noise-ratio (SNR) peaks or baseline signals [39], with slight changes in the phase factors substantially altering apparent peak heights and positions. Phasing must also often be performed manually for low SNR peaks, for which automatic phasing algorithms can be unreliable.

The AMARES algorithm is powerful and flexible in the parameterization of the models that it fits to spectral data, but can be unreliable for multi-frame dynamic spectral fitting because each spectrum in a time course is fit independently and potentially inconsistently. Applied to a single or small number of high SNR spectra, AMARES results could be carefully checked to ensure quality, but this is impractical with hundreds of separate spectra. Increasing the number of free model parameters can further increase the noisiness and inconsistency of fit results between frames. Variations in parameters, like peak width or frequency, between frames in less-constrained models also suggest that the results of the amplitude parameter fit could be unreliable or biased. A highly constrained model provides a more robust fit, but this requires approximating the fixed parameters from other peaks, summed spectra, or the previous less-constrained fits. Inconsistent area under the curve ratios results between models also make it unclear whether fit parameters were biased by the fixed values.

Because no ground truth spectra are available to validate the results in this work, the motivation for narrow bandwidth excitation is primarily the ease and robustness of its application. By removing the background from small peaks during acquisition, the complexities of correcting for them during quantification can be avoided, and linear signal averaging over time may be applied more easily, without introducing bias from flawed parametric assumptions.

Slice spectroscopy with hyperpolarized [1-^13^C]pyruvate has a wide range of applications, such as tumor characterization and therapy response monitoring in subcutaneously implanted rodent tumor models, including patient-derived xenografts [14,16]. It has also been interleaved with imaging excitations [4,15] and used to localize signal in the ipsilateral side of a brain implanted with a tumor [39] or acute myocardial ischemia [40]. In contrast, spectroscopy of hyperpolarized [1-^13^C]lactate and its metabolites has been less well studied. Long polarization times and low levels of downstream metabolites are the biggest limitations of [1-^13^C]lactate imaging; the pyruvate pool size is small and the label exchange from lactate to pyruvate is rather slow [30].

An alternative and more sensitive detection method to measure LDH-catalyzed label exchange is observation of the exchange of the C2 deuterium label between injected hyperpolarized [1-^13^C,U-^2^H]lactate and endogenous unlabeled lactate. However, this requires monitoring the phase modulation of the spin-coupled hyperpolarized ^13^C signal using a ^1^H/^13^C spin–echo experiment [41].

Lactate can be injected at a more physiologic concentration than pyruvate can, and has several potential applications including investigation of metabolic processes in skeletal muscle [31] and liver [29] as well as use as metabolic neuroprotective biosensor for ischemic stroke [6]. Recent studies identified lactate as an important metabolic fuel for spontaneously grown solid tumors in humans and rodents [42], indicating that [1-^13^C]lactate may be useful for tumor characterization [41]. Moreover, comprising evidence underlines the importance of lactate as a signaling molecule especially in the inflammatory processes. Presence of lactate regulates immune cell polarization, differentiation and growth, as well as tumor immune surveillance [12]. These findings suggest that lactate could potentially be developed as metabolic biomarker for inflammatory disease and tumor stratification, as well as therapy response monitoring.

Other metabolic systems could also benefit from improved isolation of low-level hyperpolarized ^13^C signal, including [^13^C]glucose metabolism [38] and quantification of [^13^C]HCO_3_^−^ and [^13^C]CO_2_ equilibrium for pH measurement [43] or analyses of aldehyde dehydrogenase activity in the liver with hyperpolarized [1-^13^C, U-^2^H5]ethanol [44]. In addition, metabolic systems with more than two measurable metabolite frequencies, including [1-^13^C]pyruvate imaging, can be measured with narrow bandwidth excitation. However, greater care would need to be taken in such cases due to the closer spacing of metabolite frequencies. Even narrower bandwidth—and thus longer duration—RF pulses may remain usable for ^13^C labeled compounds, due to their relatively [44] long T2* relaxation time constant in vivo. Alternatively, pre-saturation of intermediate-frequency metabolites [45] may be applied.

## 5. Conclusions

Hyperpolarized injected [1-^13^C]lactate and its metabolite [1-^13^C]pyruvate were measured with narrow bandwidth slice-selective spectroscopy, improving the quantification of the low-signal downstream metabolite. The spatial offset of slices was used to produce a variable-frequency excitation scheme, without the need for explicit pulse sequence support or implementation of advanced spectral–spatial radiofrequency and gradient pulses. This method does not require special equipment, is easy to employ, simplifies data processing for dynamic measurements, and may be beneficial for a range of hyperpolarized substrates and preclinical disease models.

## Figures and Tables

**Table 1:** Broad bandwidth excited AMARES fit peak amplitudes and broad and narrow bandwidth excited spectral magnitude area under the curve ratios (AUCr) of [1-^13^C]alanine and [1-^13^C]pyruvate over [1-^13^C]lactate (Ala/Lac and Pyr/Lac), summed starting from the frame with the initial appearance of hyperpolarized [1-^13^C]lactate signal to the end of acquired data (frames 32 to 150). Two human pancreatic cancer (PSN1) tumors were each measured with one of the methods. The corresponding data summarized here are also shown in Figure 2 and Figure 3.

	AMARES Lightly Constrained	AMARES Moderately Constrained	AMARES Highly Constrained	Magnitude Spectrum Broad BW	Magnitude Spectrum Narrow BW
**Ala/Lac**	0.021	0.020	0.027	0.058	N/A
**Pyr/Lac**	0.035	0.037	0.040	0.035	0.025

## Data Availability

The data presented in this study are available on request from the corresponding author.
